# Non-edible Oil Cakes as a Novel Substrate for DPA Production and Augmenting Biocontrol Activity of *Paecilomyces variotii*

**DOI:** 10.3389/fmicb.2017.00753

**Published:** 2017-05-02

**Authors:** Kalpana Arora, Satyawati Sharma, Suresh B. N. Krishna, Jamila K. Adam, Ashwani Kumar

**Affiliations:** ^1^Centre for Rural Development and Technology, Indian Institute of Technology DelhiNew Delhi, India; ^2^Centre for Technology and Development, Society for Economic and Social StudiesNew Delhi, India; ^3^Department of Biomedical and Clinical Technology, Durban University of TechnologyDurban, South Africa; ^4^Metagenomics and Secretomics Research Laboratory, Department of Botany, Dr. Harisingh Gour University (Central University)Sagar, India

**Keywords:** jatropha cake, karanja cake, dipicolinic Acid (DPA), *Paecilomyces variotii*, medium optimization, biocontrol

## Abstract

The present study investigated the use of waste non-edible oil cakes (Jatropha, Karanja, Neem, and Mahua) as a substrate for the growth of *Paecilomyces variotii* and dipicolinic acid (DPA) production. Previous researches proved the efficacy of DPA in suppressing certain pathogens that are deleterious to the plants in the rhizosphere. DPA production was statistical optimized by amending non-edible oil cakes in growing media as nitrogen and sugars (Dextrose, Glucose, and Lactose) as carbon source. Plackett-Burman design (PBD), indicated that Jatropha cake, Karanja cake, and Dextrose were the most significant components (*p* < 0.05) of the media and were further optimized using response surface methodology (RSM). Jatropha cake, Karanja cake, and Dextrose at the concentration of 12.5, 4.5, and 10 g/l, respectively, yielded 250 mg/l of DPA, which was 2.5 fold more than that obtained from basal medium. HPLC analysis of the optimized medium (peak at retention time of 30 min) confirmed the enhanced DPA production by *P. variotii*. The scanning electron microscopy (SEM) images showed that optimized medium impose a stress like condition (due to less C:N ratio) for the fungus and generated more spores as compared to the basal medium in which carbon source is easily available for the mycelial growth. The antimicrobial activity of the fungal extract was tested and found to be effective even at 10^−2^ dilution after 72 h against two plant pathogens, *Fusarium oxysporum* and *Verticillium dahlia*. Statistical experimental design of this study and the use of non-edible oil cakes as a substrate offer an efficient and viable approach for DPA production by *P. variotii*.

## Introduction

Previous researches proved the efficacy and adaptability of *Paecilomyces* in effectively controlling different pathogens under various environmental conditions (Dunlap et al., 2007; Anastasiadis et al., 2008; Sharma et al., 2012; Barakat and Saleh, 2016). *Paecilomyces* is morphologically characterized by having flask-shaped phialides, which is differentiated into swollen base and a distinct neck and generate conidia that are arranged end to end in a chain. *Paecilomyces* species is known to utilize a variety of substrates due to its ability to consume high ammonia and nitrogen rich substrates (Liu et al., 2016). Furthermore, produces bioactive substances such as dipicolinic acid (DPA), which has been reported to suppress certain pathogens that are deleterious to the plants in the rhizosphere (Asaff et al., 2005, 2006). Use of microbial inoculants as biocontrol agents for controlling disease and pests of crop plants is continuously gaining attention as an alternative tool against the use of chemical pesticides in developing countries (Dale et al., 2007). Very few experimental studies have been reported on the DPA production from *Paecilomyces* strain and its use as an agent for control of plant pathogens.

Reported by several workers that media constituents simply affect the virulence of biocontrol agents against plant pathogens (Cliquet and Jackson, 2005; Nisha and Kalaiselvi, 2016) and its composition plays a significant role in growing fungal mycelia with increased sporulation (Sun and Liu, 2006; Gao et al., 2007). Liu and Chen (2002) reported that fungi can use wide range of substrates as carbon and nitrogen sources. However, low cost and simple media will be a better choice for their mass-production and also for field application. For successful use of a mycopesticide in biocontrol, it is essential to produce high yields of propagules which are pathogenic toward target pathogen. The quality as well as quantity of propagules is affected by medium composition and culture conditions (Gupta et al., 2016). Carbon-Nitrogen ratio as well as type of sugar in media plays an important role in determining the pathogenicity of the fungus (Vidal et al., 1998; Gao et al., 2007; Zhao et al., 2013; Nisha and Kalaiselvi, 2016). Therefore, nitrogen and carbon sources were screened for the production of bioactive molecule because they may influence the activity of phytopathogens, and enhances antibiotics production in biocontrol strains and contribute to the variability of biocontrol in different soils and also on host crops (Milner et al., 1995; Thomashow and Weller, 1996; Cliquet and Jackson, 2005; Moorthi et al., 2015).

Previously, media optimization studies reported the use of one factor at a time for various biomolecules production which is laborious and time consuming process, it also often misleads the understanding of the system behavior, generates confusion, and variation in prediction (Singh and Chhatpar, 2010; Saharan et al., 2011; Nisha and Kalaiselvi, 2016). Plackett and Burman's design (PBD), when applied to evaluate medium components, will screen the insignificant factors out of a large number of possible factors at fairly early stages of the experiment. Box Behnken Design (BBD) is useful in determining the effect of key factors, by minimum number of experiments for further optimization (Singh and Chhatpar, 2010; Zhao et al., 2013). No published reports to the best of our knowledge are available for increased production of the bioactive DPA from *P. variotii* using response surface methodology (RSM). The findings of this study strongly support the hypothesis that better yields can be achieved by optimization of C and N sources using RSM technique.

## Materials and methods

### Microorganism and culture condition

*Paecilomyces variotii* strain employed in this study was procured from the culture collection of International Centre for Agriculturally Important Microbes (ICAIM), PUSA, New Delhi, India. The potato dextrose agar (PDA) was used to grow the fungus on plates at 28°C for 7 days and it stimulated the production of conidia. These conidia were sub-cultured under submerged fermentation (SmF) in Erlenmeyer flask (500 ml) containing 100 ml of the medium containing (per liter): Glucose (30 g), yeast extract (3 g), KH_2_PO_4_ (0.39 g), Na_2_HPO_4._12H_2_O (1.42 g), MgSO_4._7H_2_O (0.60 g), NH_4_NO_3_ (0.70 g), and KCl (1.00 g) (Fargues et al., 1992). The medium pH was adjusted to 5.6 before sterilization and inoculated with 4 ml of 10^7^ conidia/ml from conidial suspension. The flasks were then incubated at 27°C for 7 days in an incubator (Scigenics Biotech, India) and the inoculum thus prepared was used for harvesting conidial spores. The spores were collected in sterile solution (0.05% Tween 80) and 50% glycerol stocks were maintained from Tween 80 conidial suspension culture and stored at −20°C. This spore suspension was utilized as inoculums in all further experiments. For using oil cakes as media, the non-edible oil cakes i.e., Jatropha (*Jatropha curcas*), Karanja (*Pongamia pinnata*), Neem (*Azadirachta indica*), and Mahua (*Madhuca indica*) were oven dried at 55°C for 24 h, grinded, and sieved to obtain 0.8- and 2.0-mm particle size. C:N ratio in the cakes was analyzed. Oil cake was soaked in water overnight, then heated for 2 h and final volume was adjusted by adding water to prepare cake broth.

### Analytical methods

#### Biomass estimation

Gravimetric analysis was used to determine biomass in submerged fermentation as described by Soundrapandian and Chandra (2007). The liquid from flask was filtered through pre-weighed Whatman filter paper Grade 1 (pore size, 11 μm) and dried at 70°C under vacuum (0.6 atm) until reaching the constant weight, by determining the weight difference of the filter paper. Spore count was done with a Neubeaur's hemocytometer.

#### Chemical analysis of the samples

The culture filtrate was centrifuged at 10,000 rpm for 10 min and supernatant was filtered through nylon filter (pore size 0.45 μm) and divided into two parts for analysis. One part was kept for spectrophotometric analysis of DPA and second part (50-ml aliquots) was freeze-dried and then extracted using sonication with 50 ml methanol acetate at 25°C for 10 min.

#### Qualitative analysis

The extracts with methanol and ethyl acetate were used for Thin Layer Chromatography (TLC). Silica gel GF_254_ plate (E. Merck, Dramstadt, Germany) was used with 1-butanol/water/acetic acid (12:5:3, by vol.) solvents. The plates were exposed to UV light (254 nm) to visualize the bands and confirmation was done by spraying a 0.051 M cerium ammonium sulfate solution and heating on a hot plate for color development. A DPA standard (Sigma) (Rf 0.90) was used for determining the presence of DPA (Asaff et al., 2005).

High-Performance Liquid Chromatography (HPLC) analysis of the collected samples were performed using a Thermo-separation (Constametric 4100) chromatograph using following conditions: UV detector and a Phenomenex OOH-0138-KO column, temp 30°C, mobile phase 5 mM H_2_SO_4_, flow rate of 0.6 ml/min and 210 nm wavelength for compound detection (Asaff et al., 2005).

### DPA estimation

DPA content was quantified using a UV-visible spectrophotometer (Asaff et al., 2006). Sample (0.8 ml) to be quantified was mixed with 0.2 mL of ferrous ammonium sulfate (1 g) and ascorbic acid (1 g) and dissolved in 100 ml of 0.5 M acetate buffer and with a final pH of 5.5. Absorbance was measured at 440 nm wavelength in UV-visible spectrophotometer (PerkinElmer model Lambda 25 UV/VIS, Massachusetts, USA).

### Experiment design

Culture medium was optimized using non-statistical as well as statistical experimental designs. Medium components were selected by using one-factor-at-a-time approach. The preliminary experiments revealed the optimal range of carbon (Glucose, Dextrose, and Lactose) and nitrogen sources (Jatropha oil cake, Karanja oil cake, Neem oil cake, and Mahua oil cake) and its effect on sporulation and DPA production by the fungus (Table 1). This data was utilized for designing further experiments using PBD.

**Table 1:** **Growth and bioactive substance (DPA) production by ***P. variotii*** on non-edible oil cakes used as solid medium and broths (after 10 days)**.

**Non-edible oil cake**	**Treatment**	**Solid medium**		**Broth**		
		**Growth/day (cm/day)**	**Spore count (spores/g of substrate)**	**Dry mycelial weight (g/100 ml)**	**Spore count (spores/ml)**	**DPA production (mg/l)**
C1	Alone	0.42 ± 0.01^c^	6.7 × 10^9^	0.29 ± 0.04^d^	2.7 × 10^9^	102 ± 3.5
	+ PDA (1:1)	0.35^c^	9.0 × 10^9^	0.15 ± 0.016^a^	7.9 × 10^8^	110 ± 1.2
	+ dextrose	0.36^d^	5.1 × 10^10^	0.31 ± 0.02^d^	3.4 × 10^9^	135 ± 2.3
C2	Alone	0.44^d^	5.1 × 10^9^	0.271 ± 0.02^d^	2.0 × 10^9^	70 ± 1.2
	+ PDA (1:1)	0.32 ± 0.02^d^	6.2 × 10^9^	0.135 ± 0.005^a^	3.1 × 10^8^	78 ± 3.5
	+ dextrose	0.23 ± 0.01^c^	2.9 × 10^10^	0.28 ± 0.04^d^	1.9 × 10^9^	83 ± 1.3
C3	Alone	0.25 ± 0.02^d^	4.5 × 10^9^	0.275 ± 0.02^d^	2.1 × 10^9^	108 ± 2.1
	+ PDA (1:1)	0.31 ± 0.02^d^	1.0 × 10^9^	0.13 ± 0.01^a^	3.0 × 10^8^	116 ± 1.1
	+ dextrose)	0.35 ± 0.01^d^	2.1 × 10^10^	0.28 ± 0.04^d^	1.8 × 10^9^	136 ± 2.5
C4	Alone	0.18 ± 0.015^b^	7.1 × 10^8^	0.20 ± 0.03^b^	10 × 10^7^	60 ± 3.4
	+ PDA (1:1)	0.18 ± 0.01^c^	8.3 × 10^7^	0.12 ± 0.04^a^	8.7 × 10^7^	52 ± 1.8
	+ dextrose)	0.20^c^	7.7 × 10^7^	0.18 ± 0.01^b^	6.5 × 10^7^	45 ± 3.4
Control	PDA (Potato Dextrose Agar)	0.26 ± 0.01^c^	2.5 × 10^9^	0.249 ± 0.01^c^	1.5 × 10^9^	91 ± 1.0

*C1, Jatropha oil cake; C2, Neem oil cake; C3, Karanja oil cake; C4, Mahua oil cake. Same letter used in each column indicates insignificant difference among the treatments (p < 0.05) according to DMRT. Value are means of n = 4*.

All experiments were performed in triplicate in 500 ml Erlenmeyer flasks with 100 ml working volume. The average crude DPA concentration and spore count were taken as the dependent variables or response (Y). A correlation was also determined between production of DPA and spore of the fungus.

### PBD for identification of the significant variables

To increase DPA production, carbon and nitrogen sources were optimized using PBD. PBD screen “n” variables in just a minimum of “n + 1” number of experiments (Plackett and Burman, 1946). It was used to identify the relatively important medium constituents i.e., C and N sources. PBD design evaluates the impact of main factors, as well as their interaction in production of DPA and produce optimal or near optimal responses. A total of seven factors were studied as described in Table 2 including their low (−1) and high levels (+1). The PBD was based on the first order model:

(1) $$\begin{array}{r} Y=\beta_{0}+\sum \beta_{i}X_{i} \end{array}$$

Where *Y* is the response (DPA production), β_0_ is the model intercept, and β_*i*_ is the variable estimates. The factor level with confidence level above 95% is considered the most significant factor that affects the DPA production. A 25 PBD leading to 30 sets of experiments were performed in triplicate to verify the most significant factors affecting the DPA production, as shown in Table 3.

**Table 2:** **Levels of various Carbon and Nitrogen sources tested in Plackett-Burman Design (PBD)**.

**Variables code**	**Independent Variables**	**Levels (g/l)**	
		**Low level −1**	**High level +1**
x_1_	Glucose	15	60
x_2_	Dextrose	15	60
x_3_	Lactose	15	60
x_4_	Karanja	10	40
x_5_	Jatropha	12	48
x_6_	Neem	12	48
x_7_	Mahua	17	56

**Table 3:** **PBD and results of the fractional factorial design**.

**Run**	**x_1_**	**x_2_**	**x_3_**	**x_4_**	**x_5_**	**x_6_**	**x_7_**	**DPA (mg/l)**	
								**Predicted**	**Observed**
1	−1	1	1	−1	−1	−1	−1	89.4	88.2
2	−1	1	−1	−1	1	−1	−1	90.1	94.3
3	1	1	−1	−1	−1	1	−1	79.7	78.2
4	1	1	−1	−1	−1	−1	1	89.6	90.2
5	−1	−1	−1	1	1	1	−1	90.9	91.2
6	−1	−1	−1	1	−1	−1	1	88.6	88.6
7	−1	−1	−1	1	1	1	1	96.2	94.6
8	1	−1	1	1	1	−1	1	93.1	94.2
9	1	−1	−1	1	1	−1	−1	140.1	138
10	−1	−1	1	−1	−1	1	−1	96.2	98.4
11	−1	1	−1	−1	−1	1	1	99.4	100.6
12	−1	−1	1	1	1	−1	−1	104	105.4
13	−1	−1	1	−1	−1	−1	1	96.8	96.8
14	−1	1	−1	1	1	1	−1	152	155
15	1	1	1	1	1	−1	−1	94	91.2
16	1	−1	1	1	−1	−1	−1	95.2	94.2
17	1	−1	−1	−1	−1	1	1	97.3	98.8
18	−1	1	1	1	−1	−1	1	130.2	132
19	−1	1	1	−1	1	−1	1	93.3	94.4
20	1	−1	1	−1	1	−1	−1	91	89.8
21	−1	1	1	−1	1	1	−1	98.2	97.8
22	1	1	−1	1	−1	−1	−1	99.7	100.4
23	1	1	−1	−1	1	1	1	93	92.2
24	−1	−1	−1	−1	1	1	−1	93	92.3
25	−1	−1	1	1	−1	1	1	100	99.6
26	1	1	1	1	1	1	1	68.7	70.3
27	1	1	−1	1	−1	1	1	88.3	88.6
28	1	1	1	−1	−1	1	1	92.4	93.2
29	1	−1	−1	−1	1	−1	1	92	91.2
30	1	−1	1	1	1	1	−1	92.4	93.6

### Optimization of the selected trace concentration by response surface methodology (RSM)

RSM is an efficient method to answer multivariate problems and optimizing several responses by minimizing the number of experiments (Kumar et al., 2016). Box Benkhen design (BBD) and PBD are part of RSM. In this experiment PBD was used initially to select the components affecting DPA production and BBD was used to optimize the major variables designated as x_1_, x_2_, and x_3_. Seventeen experimental runs were generated by the software comprising of different combinations of three factors. A second-order polynomial function was fitted to correlate the relationship between independent variables and response for predicting the optimal point. For three factors this equation is

(2) $$\begin{array}{r} Y_{pred}=\beta_{o}+\sum \beta_{i}x_{i}+\sum \beta_{ij}x_{j}^{2}+\sum \beta_{ij}x_{i}x_{j} \end{array}$$

Where, Y_*pred*_ is the predicted response, xi and x_*j*_ are the input variables which influence the response Y. β_*i*_ is the i^*th*^ linear coefficient, β_*ij*_ is the ij^*th*^ interaction coefficient and β_*o*_ is the constant. A quadratic polynomial equation was used to fit the data obtained.

### Software for experimental design

The “Design-Expert” software version 7.0 (Stat-Ease, Inc., Minneapolis, USA) was used for the construction and evaluation of the statistical experimental design. The RSM contour plot analysis provides the optimal C and N values for DPA production.

### Preparation of *P. variotii* samples for morphological study

The *P. variotii* cells were harvested by centrifugation from the run showing a maximum yield of DPA and control after the completion of BBD. The harvested cells were prefixed with 2.5% glutaraldehyde solution overnight at 4°C. The cells were centrifuged and washed thrice with 0.1 M sodium phosphate buffer (pH 7.2) and serial dehydration of the cells was done using different gradient of ethanol. Later, the cells were dried at “critical point” (Tyagi and Malik, 2010).

For SEM standard protocol was used to study the morphology of the *P. variotii* cells under SEM (ZEISS EVO 50, Germany). The SEM images were clicked using following parameters: EHT = 20.00 kV, WD = 12.0 mm, Signal A = SE1.

### Biocontrol activity *in vitro*

The antagonistic activity of the *P. variotii* against two phytopathogenic fungi *Fusarium oxysporium* and *Verticillium dahilae* was studied by dual culture test by following the method of Rahman et al. (2009). *P. variotii* was inoculated on both the sides of Potato dextrose Agar (PDA) plate which contained 6 mm disc of pre-grown phytopathogenic fungi at the center. The biocontrol activity of *P. variotii* against the fungal pathogens was observed in terms of measuring the diameter of zone of inhibition. Phase Contrast Microscope (Leica Model, DME, Germany) at 10 and 40X magnification after 2–5 days of incubation at 28°C was used for identification of fungal mycelium along the edges of the inhibited colonies facing *P. variotii*.

## Results and discussion

Carbon and nitrogen are known to be most essential nutrients required by fungi for their growth. C:N ratio in the medium is important since it significantly affects the spore yield of the bioherbicidal fungi (Engelkes et al., 1997; Gao et al., 2007). Media having 15:1 C:N ratio promote more conidial production along with biocontrol efficacy. Results showed that C:N ratio is more important for the sporulation of *P. variotii*. The cakes of Jatropha, Karanja, Neem, and Mahua were found to have C:N ratios of 2.76, 2.29, 3.28, and 6.3 respectively.

### PBD for the screening of important factors for DPA production

Seven medium elements which have the possibility to influence the production of active substance, namely Glucose, Dextrose, Lactose (carbon sources), and Jatropha oil cake, Karanja oil cake, Neem oil cake, and Mahua oil cake were selected for a 2-level PBD. The predicted regression equation for the DPA (Y) production as a function of the coded values of critical variables is given in the following equation:

(3) $$\begin{array}{l} Y=98.68-6.09x_{1}+5.09x_{2}-3.97x_{3}+6.75x_{4}+4.53x_{5}\\ \;-\;0.97x_{6}-1.47x_{7}-9.70x_{1}x_{2}-5.39x_{1}x_{3}-5.6139x_{1}x_{4}\\ \;-\;3.53x_{1}x_{6}-0.84x_{1}x_{7}+3.94x_{2}x_{4}+4.92x_{2}x_{5}+1.27x_{2}x_{7}\\ \;+\;4.30x_{3}x_{7}+5.67x_{4}x_{5}-3.32x_{4}x_{6}+1.57x_{5}x_{6} \end{array}$$

From Equation (3) it can be observed that Karanja oil cake (x_4_), due to its higher coefficient effect was the most significant factor. Although, the results have also given the picture that the Jatropha oil cake and dextrose are also necessary in certain amounts to switch on the maximum production of DPA. These factors contain some important elements that may influence the activity of some enzymes (Deqing, 2002; Saharan et al., 2011) and some enzymes included in non-ribosomal peptide synthetase (NRPSs). The biochemical mechanism related to the yield of DPA by non-edible oil cakes needs further research. The results of *F*-test showed significant difference (*p* < 0.01) for variance between the average of observation of two-level experiment and center point. The result revealed that the optimum point was in accordance with the design used in the experiment.

### Optimization of selected C & N sources by RSM using BBD

Screening of the factors helped in the detection of gross curvature in the design space. The BBD was applied to estimate quadratic effects, pure process variability and reassess gross curvature, with active substances produced as response (Wang and Liu, 2008). Based on the variable identified by the PBD, a 3-level BBD was developed for variables significantly affecting DPA production (Table 4). The BBD of the three screened variables in coded format, along with DPA concentration as responses is depicted in Table 5. The maximum yield of DPA production, experimentally observed, was 250 mg/l in run 12. In order to get the optimum values of the variables which correspond toward maximum DPA production, a second-degree polynomial model was proposed. By applying a second-degree polynomial equation (Equation 4) in multiple regression analysis, explains the function of each variable and their interactions in the production of active substance:

(4) $$\begin{array}{l} Y_{pred}=341.2+47.0x_{1}+119.6x_{2}+225.5x_{3}-2.66x_{1}^{2}-13.1x_{2}^{2}\\ \;-\;97.1x_{3}^{2}+0.37x_{1}x_{2}+2.83x_{1}x_{3}-6.16x_{2}x_{3} \end{array}$$

Where Y is the predicted active substance (DPA) yield, x_1_ is Dextrose, x_2_ is Karanja oil cake, and x_3_ is Jatropha oil cake.

**Table 4:** **Coded and real values for Box Behnken design (concentration g/l)**.

**Variables**	**Level of variables**		
	**−1**	**0**	**1**
Dextrose	5	10	15
Jatropha	1	2	3
Karanja	2	4	8

**Table 5:** **Response from Box Behnken design experiment (conc. g/l)**.

**Run**	**x_1_**	**x_2_**	**x_3_**	**DPA (mg/l)**
1	−1.000	0.000	−1.000	172.5
2	0.000	0.000	0.000	251.2
3	0.000	0.000	0.000	249.8
4	1.000	0.000	1.000	107
5	0.000	0.000	0.000	250.4
6	−1.000	0.000	1.000	138
7	−1.000	1.000	0.000	155
8	0.000	−1.000	1.000	130
9	1.000	0.000	−1.000	99.2
10	0.000	−1.000	−1.000	140
11	1.000	1.000	0.000	115
12	0.000	0.000	0.000	248.2
13	0.000	0.000	0.000	253
14	−1.000	−1.000	0.000	140
15	0.000	1.000	1.000	128
16	0.000	1.000	−1.000	175
17	1.000	−1.000	0.000	115

In first order, main effect of Jatropha oil cake (*p* = 0.0501) and Karanja oil cake (*p* = 0.0045) are less significant than their quadratic main effect (*p* < 0.001) indicating the effect of Jatropha and Karanja oil cake in DPA production (Table 6). Similarly, the interaction of Dextrose—Jatropha (*p* < 0.0001) and Dextrose—Karanja (*p* < 0.0001) and the quadratic are also quite significant among all interactions. Results of second order response surface model using ANOVA (analysis of variance) are shown in Table 7. The low probability value obtained from Fisher's *F*-test [(*P* model > F) < 0.0001] showed the high significance of the model. The coefficient (R^2^ = 0.9833) described the fitness of the model, which ascertained that > 98% sample variation was ascribed to the variables and only <2% of the total variance could not be explained by the model (Table 7). The model significance was also justified by the adjusted determination coefficient (Adj *R*^2^ = 0.9847) and predicted determination coefficient (0.8962). The statistically insignificant lack of fit was also shown by model [(*P* model > F) = 0.22], making the model adequate for prediction within the range of variables employed. A coefficient variation having low value (CV = 4.26%) proposed the preciseness and reliability of experiments. The linear pattern in the normal plot of residuals, demonstrated normality in the error term, i.e., there were no indications of any problem in the data (Draper and Smith, 1981). The correlation coefficient of 0.94 reveals the strong positive correlation between the both spore formation and DPA production. Increase in the number of spore's leads to more production of DPA by the fungus.

**Table 6:** **Significance of quadratic model coefficients for DPA production**.

**Factor**	**Coefficient values**	***F*-values**	***p*-value**
Intercept	341.2	115.35	<0.0001
x_1_	47.0	69.50	<0.0001
x_2_	119.6	5.59	0.0501
x_3_	225.5	16.99	0.0045
${\text{x}}_{1}^{2}$	−2.66	1.09	0.3309
${\text{x}}_{2}^{2}$	−13.1	8.68	0.0215
${\text{x}}_{3}^{2}$	−97.1	6.64	0.0367
x_1_x_2_	0.37	363.08	<0.0001
x_1_x_3_	2.83	225.96	<0.0001
x_2_x_3_	−6.16	244.14	<0.0001

**Table 7:** **ANOVA for response surface quadratic model obtained for DPA production**.

**Source**	**Sum of Squares**	**Mean Square**	**DF**	***F*-value**	***P* > *F***
Model	53514.61	5946.07	9	115.35	<0.0001
Residual	360.85	51.55	10		
Lack of Fit	295.32	49.22	6	3.004	0.22
Pure Error	*65.53*	16.382	4		
Total	53875.46		19		

*CV % = 4.26; R^2^ = 0.9833; Adj R^2^ = 0.9847; Predicted R^2^ = 0.8962*.

*Value of “p >F” less than 0.05 indicate model terms are significant*.

*The CV value >4 indicates adequate precision in the model*.

Table 7 inferred the significance of each coefficient which was determined by *F*-test and *P*-value. The greater the magnitude of *F*-value the lesser the *p*-value, the more significant is the corresponding coefficient. The generated response surfaces of three variables tested expressed a linear effect on the response (*p* < 0.001). Dextrose, Karanja, and Jatropha showed a positive effect on the production of bioactive substance.

**Figure 1:**
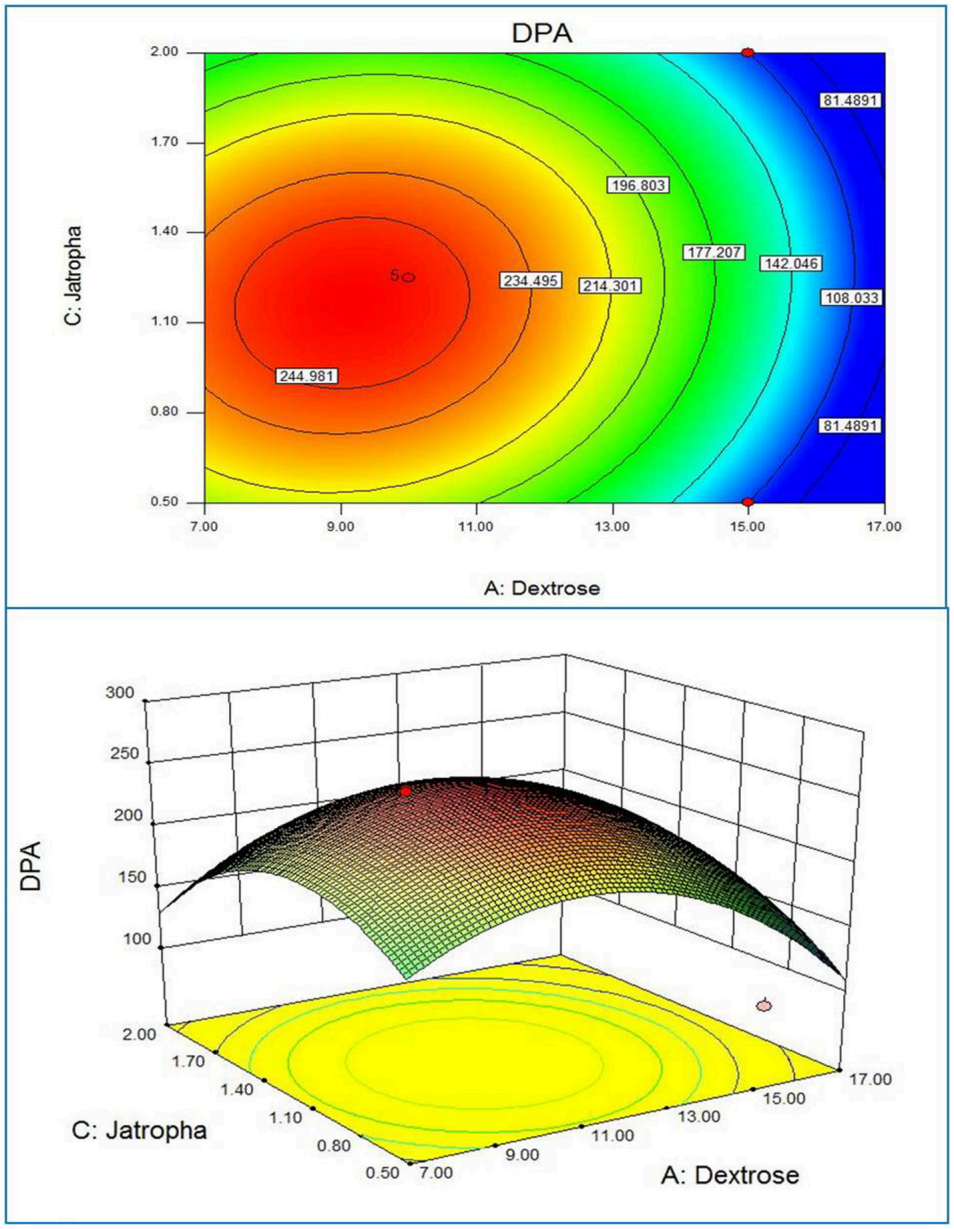
**2D contour plot and 3D response surface curve of Jatropha oil cake and Dextrose predicted by the full quadratic model**.

**Figure 2:**
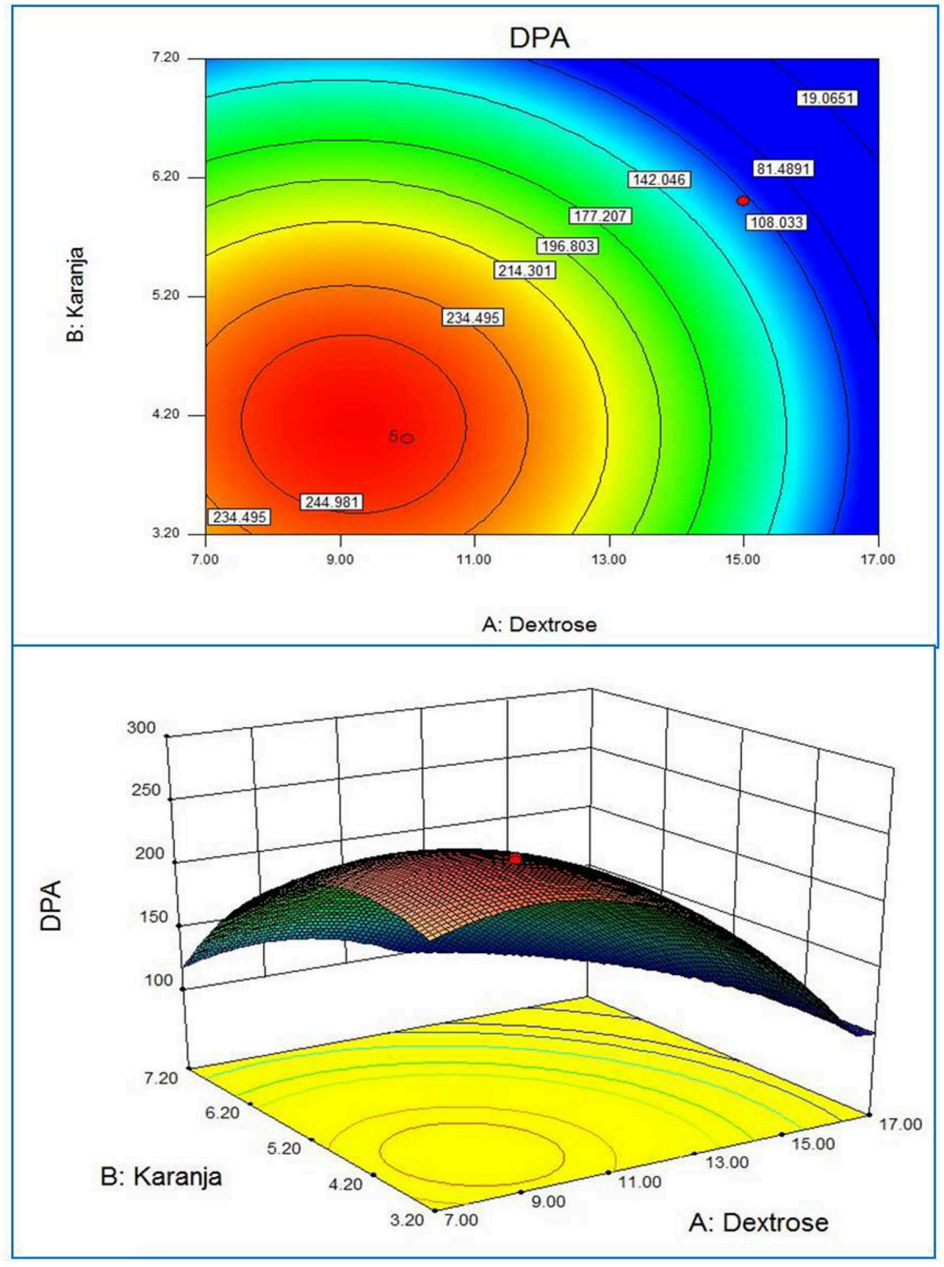
**2D contour plot and 3D response surface curve of Karanja and Dextrose predicted by the full quadratic model**.

**Figure 3:**
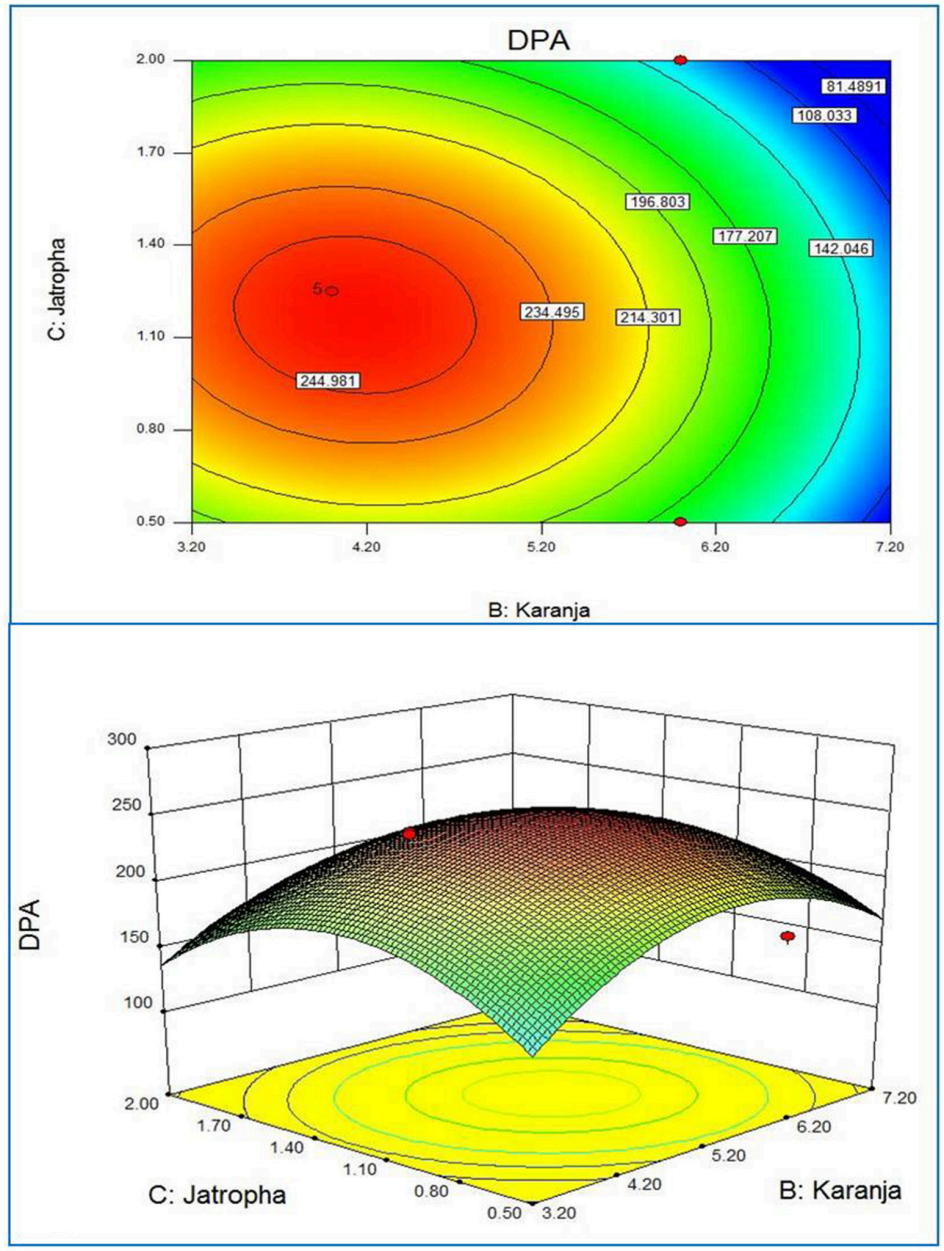
**2D contour plot and 3D response surface curve of Karanja and Jatropha predicted by the full quadratic model**.

Response surface plots of the model generated by the software gave the visualization of the role of the independent variables on the dependent one (Xu et al., 2008). The RSM plot presents a system to foresee the yield of active substances for various values at different test variables and the contours of the plot generated are useful in recognition of the kind of interaction lies in test variables. The contour plots generated by using the fitted quadratic polynomial equation obtained from regression analysis are shown in Figures 1–**3**. Each figure shows the effect of two independent variables on the active substances production, while the other variable was held at zero level. The circular contours of the plot surfaces implicate the negligible interaction between the corresponding variables whereas an elliptical or saddle contours indicates the significant interactions between the related variables (Murthy et al., 2000). The effect of *Jatropha* oil cake and dextrose on the DPA production at the fixed Karanja oil cake level is shown in Figure 1. Similarly, the Figures 2, 3 correspond to the effect of two variables on the production of DPA, holding the 3rd variable at zero level. All three cases, revealed a clear optimal convergence. The optimal levels provided three independent variables on DPA production. A significant interaction was noted between the carbon source dextrose and the nitrogen source Karanja and Jatropha oil cakes. The C:N ratio directly influenced the growth of the fungus and accumulation of the metabolites (Ramachandran et al., 2007; Abd EL-Aziz et al., 2015). Although the actual situation may be more complex than explained, an attempt for the medium optimization has been made by RSM.

### Validation of the predicted concentration in the optimal medium

Based on medium optimization, the maximum production of DPA was 250 mg/l predicted by quadratic model when the dextrose, Karanja oil cake, and Jatropha were at 10, 40, and 12.5 g/l, respectively. Validation experiment was performed in a 5 l fermentor and compared with the predicted data from the model to verify the predicted results. Fermentor experiment was done only for validation of the results obtained from the batch fermentation and was carried out in exactly same conditions. The volume was increased from 100 ml to 5 l. Scale up experiment with 5l fermentor was done at 27°C and 150 rpm (Scigenics Biotech, India) for 7 days. In the 5 l fermentor, the average concentration of DPA in the broth was 373.5 mg/l, showing 50% increase in DPA yield, suggesting that the accuracy of the model is over 95%. Where as, the predicted value for DPA by the model was 251 mg/l which translates into a deviation of only 4.64%. The level of DPA production obtained using optimized carbon and nitrogen sources amendment was 2.5 folds higher than the non-optimized basal medium for fungus. This is the first report on DPA production optimization using RSM designs in synthetic medium.

### Morphological alteration of *P. variotii*

**Figure 4:**
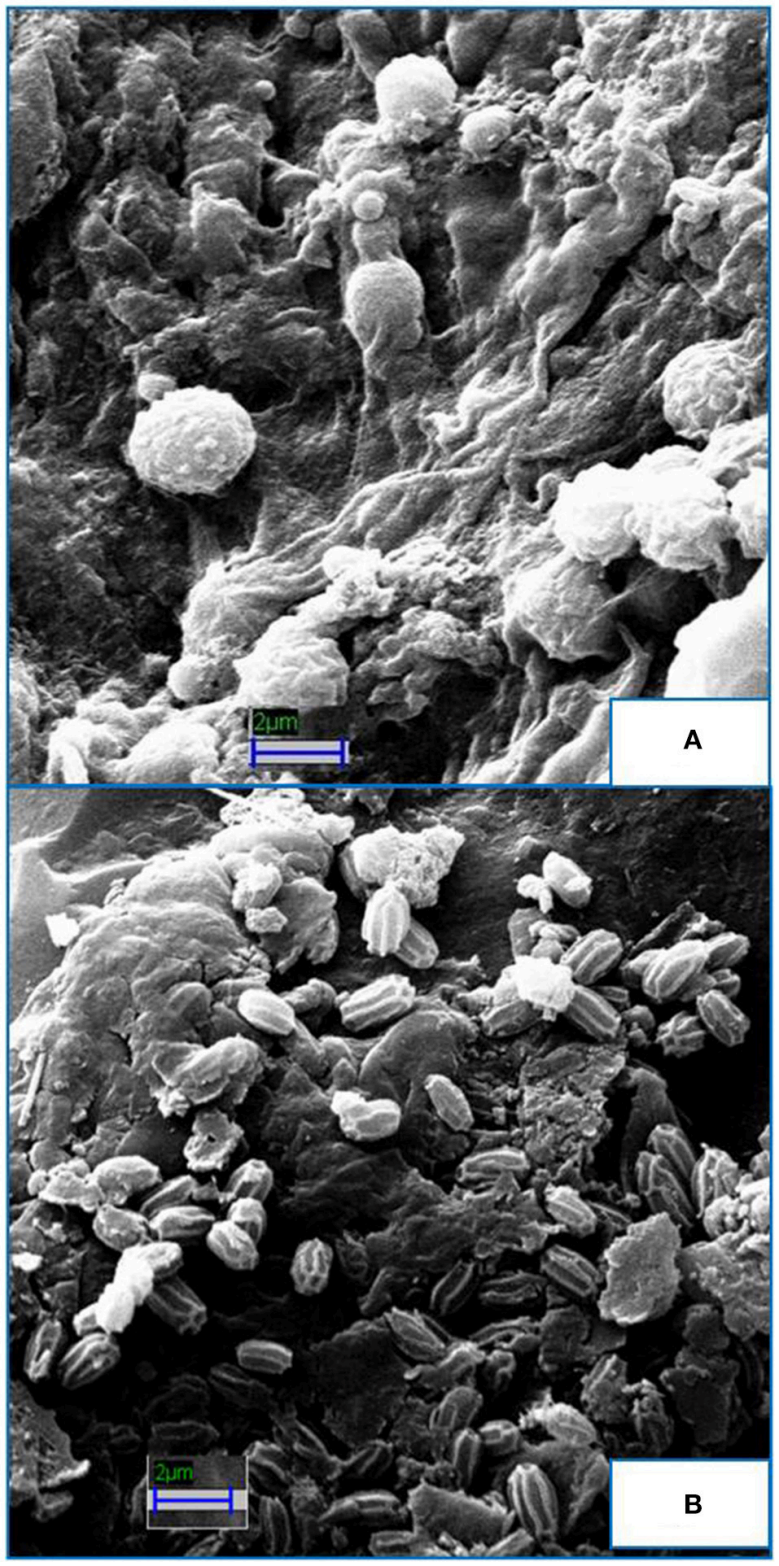
**Scanning Electron Micrographs of ***P.variotii*** spores grown in basal medium and optimized non-edible oil cake medium. (A)** Spores in normal PDB are less in number, spherical, and with smooth surface. **(B)** Spores in optimized non-edible oil cake medium are numerous, oval shaped, and ornamented surface.

SEM images show the morphological changes in *P. variotii* spores grown in basal medium and in the oil cake (Figure 4). Spores in the best-selected concentration of oil cakes (Jatropha, Karanja) underwent morphological alterations considerably in comparison to the control when observed under SEM (Figure 4). Control spores are spotted round and spherical and less in number, also there is more mycelial content (Figure 4A) while the oil cakes treated spores are numerous and oval shaped having ornamented rough surface (Figure 4B).

### Biocontrol activity

Production of bioactive substance establishes that *P. variotii* confirmed strong inhibition in dual culture after 2–5 days of incubation against *F. oxysporium* and *V. dahilae*. On further incubation, the pathogenic fungal mycelia (*F. oxysporium* and *V. dahilae*) growing toward the interacting zone inhibited and continuously lost vigor due to degradation. *P. variotii* mycelium was grew faster and covered the most part of the petriplate. It showed strong inhibition activities *in vitro* against above mentioned phytopathogenic fungi tested.

**Figure 5:**
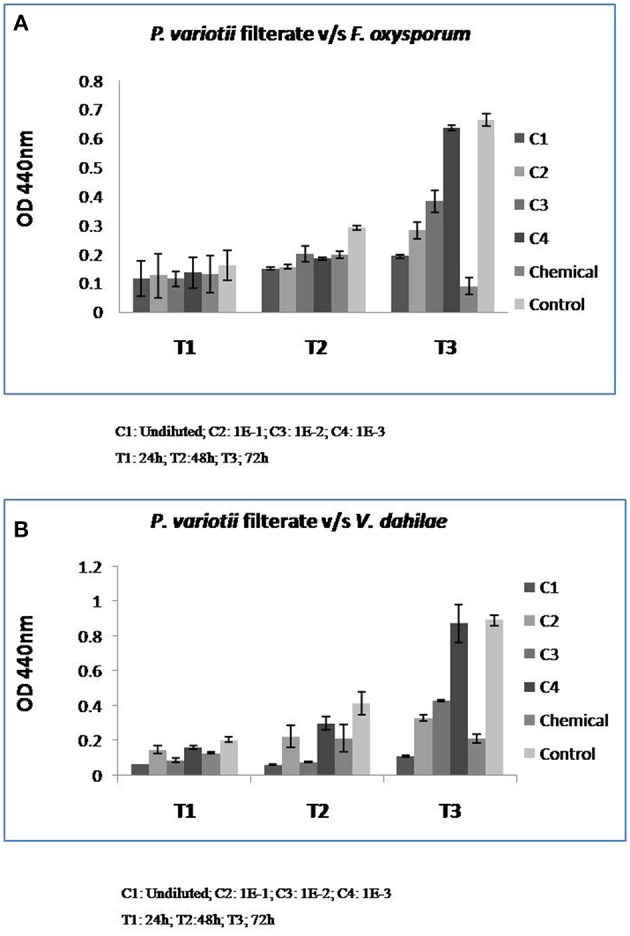
**Minimum Inhibitory Concentration (MIC) analysis of ***P. variotii*** filtrate against *F. oxysporum* (A)** and *V. dahilae* **(B)**.

The fungistatic and fungicidal concentrations of *P. variotii* extract contains DPA as observed by spectrophotometric assay are presented in Figure 5. Fungal filtrate exhibited the strong antimicrobial effect even at very low concentrations. The Minimum Inhibitory Concentrations (MICs) against both the pathogens (*F. oxysporum* and *V. dahilae*) was identified at the concentration C3 i.e., 1E-2 dilution of the filtrate (Figures 5A,B).

This study is novel in terms of optimization of media for DPA production by *P. variotii*, a low cost raw material such as non-edible oil cakes and in studying biocontrol of plant pathogens by the fungal extract. Studies and field trials proved that *P. variotii* is potential in controlling *Fusarium* and *Verticillium* wilt of tomato was aided by production of various bioactive compounds such as DPA, oxalic acid, protease, etc. The plants that were applied with DPA and fungal extract of *P. variotii* were tolerant to the severe attack of *Fusarium* and *Verticillium* wilt pathogens. Biopesticidal applications involve high amount of bioactive components which can be attained by medium components optimization for exploiting a low cost material such as non-edible oil cakes (NEOCs) (Abd EL-Aziz et al., 2015). Use of NEOCs in biodiesel sector for the production of DPA could be a solution for oil cakes disposal, since these cakes are toxic and cannot be used as animal feed like other edible oil cakes. Several factors predominantly medium constituents are known to influence production of DPA. Particularly C and N are significant for the production of these bioactive compounds. Ping (2000) and Ramachandran et al. (2007) have reported, various industrial applications of oil cakes in biotechnological and fermentation processes and their value addition by implementation in feed and energy source as well. Sharma et al. (2012) in their study used the non-edible oil cakes as a potential substrate for *Paecilomyces lilacinus* and its application as biopesticidal agent against termites. PBD was useful in recognizing *Jatropha* oil cake, Karanja oil cake, and Dextrose as vital factors affecting DPA production by *P. variotii*. No earlier studies are available on the effect of NEOCs on DPA production by *P. variotii*. Furthermore, the interaction effect among *Jatropha* oil cake, Karanja oil cake, and Dextrose was highly significant in enhancing the DPA production by *P. variotii*. The scaling up in a 5 l fermentor in an optimized medium promote enhanced DPA production by 50%. Like other metabolites maximum DPA production was also attained in the stationary phase. Our results are in agreement with Azeredo et al. (2004) and Singh and Chhatpar (2010), who observed that maximum enzyme production, obtained at the stationary phase of growth.

**Figure 6:**
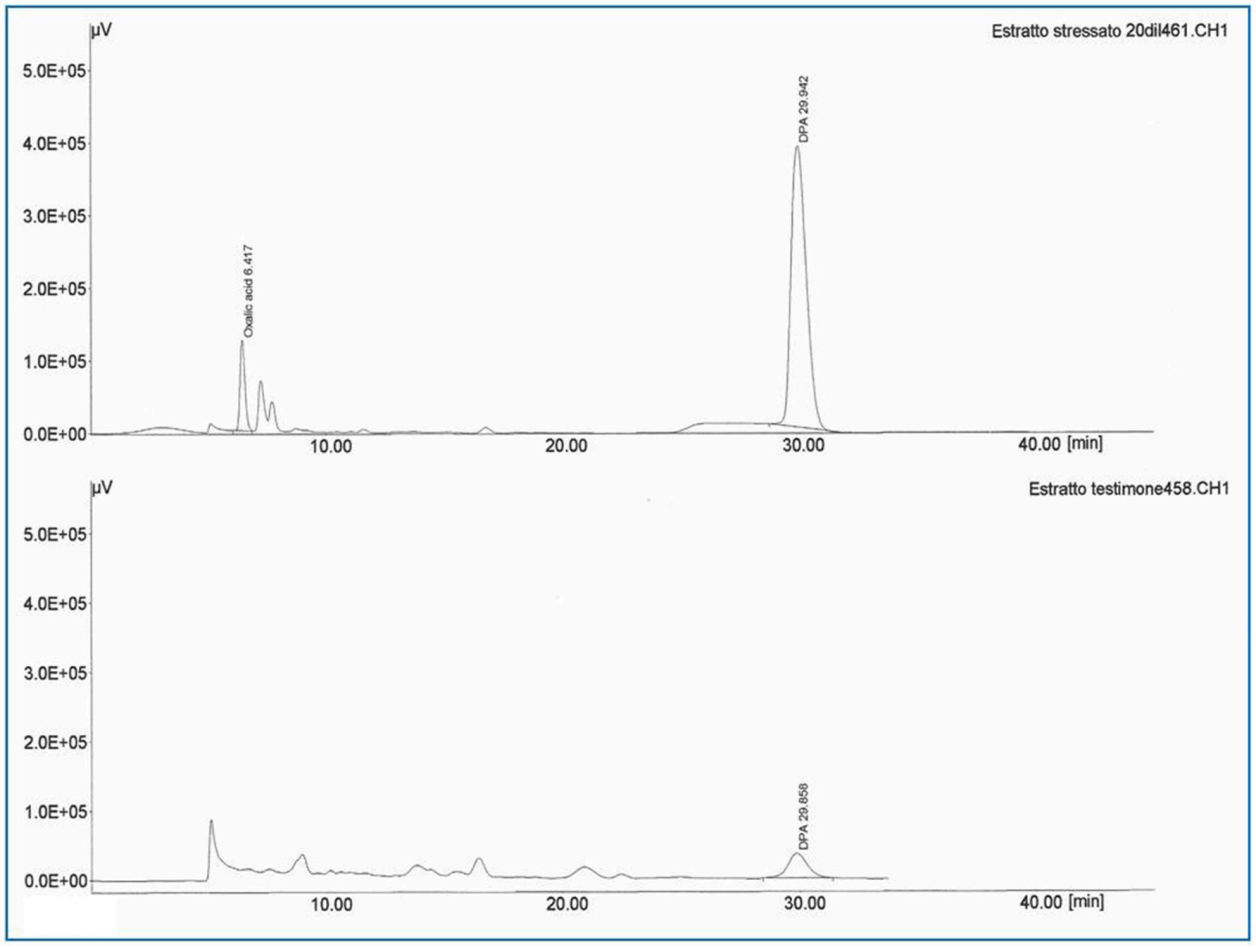
**High-Performance Liquid Chromatography (HPLC) for DPA production of control and best NEOCs (Karanja oil cake + Jatropha oil cake + Dextrose) treatment (at Retention Time 30 min)**.

The HPLC analysis of the optimized medium also confirmed the enhanced DPA production by *P. variotii* (Figure 6). The SEM analysis showed that oil cakes create a stress like condition (due to less C:N ratio) for the fungus and it generated more spores as compared to basal medium in which carbon source is easily available for the mycelial growth. The dramatic change in the morphology of the fungal cells has been observed. These results are supported by our previous observations of the DPA production during sporulation (unpublished data). During the biocontrol experiment at laboratory level the fungal extract (containing DPA) was found to be effective against *F. oxysporum* and *V. dahilae* even at low concentrations. This might be due to the Pyridine and amide moieties present in DPA. The antimicrobial activity of DPA might be due to the presence of the amide linkage groups and nitrogen heterocyclic rings, which enhances their activity (Al-Salahi et al., 2010). Moreover, the plate studies revealed the absence of any direct contact between *P. variotii* and the pathogenic fungi. This suggested that the inhibition of phytopathogenic fungi was due to exocellular, bioactive substance that diffuse through the agar medium, which substantiates the production of DPA and other bioactive substances that might inhibit the pathogen growth (Mohammadi et al., 2016). Visual observation under a phase contrast microscope, showed that the pathogenic fungal mycelia growth toward the *P. variotii* were thin and that the cytoplasmic contents of the cell became collate. However, on the control petriplate, pathogenic fungal mycelia showed regular radial growth. The results of the current work correlates with the studies conducted by Wang and Liu (2008), which optimized the medium for antifungal substance from *Paenibacillus* sp.

This study indicated that statistical experimental design recommends an efficient and realistic methodology for optimizing the requirements for DPA production by *P. variotii*. The maximum DPA production (251 mg/l), was 2.5 folds more when compared to the basal medium, achieved by optimizing carbon and nitrogen sources. This study will also contribute toward improving the DPA production by other microorganisms and the usage of trace elements. Integrated into a broader study of carbon and nitrogen sources on the production of DPA as bioactive substance, this work should help to build more rational control strategy, possibly involving scale-up production of DPA.

## Author contributions

KA performed the experiments, applied RSM, and prepared the manuscript. SS designed the experiments and reviewed the manuscript. AK, SK, and JA edited and revised the manuscript.

### Conflict of interest statement

The authors declare that the research was conducted in the absence of any commercial or financial relationships that could be construed as a potential conflict of interest.
